# Parallel synthesis of donor-acceptor π-conjugated polymers by post-element transformation of organotitanium polymer

**DOI:** 10.1080/15685551.2023.2233228

**Published:** 2023-07-06

**Authors:** Yoshimasa Matsumura, Alvin Tanudjaja, Mizuki Fukushima, Makoto Higuchi, Shin Ogino, Makoto Ishidoshiro, Yasuyuki Irie, Hiroaki Imoto, Kensuke Naka, Ryoyu Hifumi, Shinsuke Inagi, Ikuyoshi Tomita

**Affiliations:** a Department of Chemical Science and Engineering, Graduate School of Materials and Chemical Technology, Tokyo Institute of Technology, Yokohama, JAPAN; b Department of Applied Chemistry, Faculty of Engineering, Osaka Institute of Technology, Osaka, JAPAN; c Graduate School of Science and Technology, Kyoto Institute of Technology, Kyoto, JAPAN

**Keywords:** Organometallic polymers, Donor-acceptor π-conjugated polymers, Stannole, Phosphole, Arsole

## Abstract

The donor-acceptor type π-conjugated polymers having heterole units were prepared by the reaction of a regioregular organometallic polymer having both reactive titanacyclopentadiene and electron-donor thiophene-2,5-diyl units in the main chain with electrophiles such as diphenyltin dichloride, dichlorophenylphosphine, and diiodophenylarsine. For example, a polymer having electron-accepting phosphole unit was obtained in 54% yield whose number-average molecular weight (*M*_n_) and molecular weight distribution (*M*_w_/*M*_n_) were estimated as 3,000 and 1.9, respectively. The obtained polymer exhibits a high highest occupied molecular orbital (HOMO) and low lowest unoccupied molecular orbital (LUMO) energy levels (−5.13 eV and −3.25 eV, respectively) due to the electron-donating thiophene and electron-accepting phosphole units. Reflecting upon the alternating structure of thiophene and phosphole, the polymer exhibits a band gap energy level (*E*_g_) of 1.78 eV which is narrower than that of a derivative of poly(thiophene) (*E*_g_ = 2.25 eV).

## 1. Introduction

π-Conjugated polymers are important functional materials for various optoelectronic device applications, such as organic solar cells (OSCs), light-emitting diodes (LEDs), field effect transistors (FETs), and chemical sensors [1–7]. For example, narrow band gap π-conjugated polymers have great potentials for OSCs because of their effective nature of the solar energy harvesting [6,7]. π-Conjugated polymers consisting of the electron-donating and electron-accepting alternating sequences are well-known macromolecular structures for narrow band gap materials through the intramolecular charge transfer (ICT) mechanism [8–15]. To date, various ICT π-conjugated polymers that exhibit excellent optoelectronic features have been prepared mostly by the polycondensation processes based on the transition metal-catalyzed coupling reactions of monomers possessing the corresponding electron-donor and electron-acceptor units [16,17]. Although they are powerful tools to obtain versatile functional ICT π-conjugated polymers, they often constrain us to carry out multi-step monomer syntheses. The polycondensations might also be prohibited by the functional groups attached to the monomers.

As an alternative synthetic approach for functional π-conjugated polymers, we have been working on the synthesis and transformation reactions of reactive organometallic polymers. Especially, polymers containing titanacyclopentadiene-2,5-diyl units, which were prepared by the regiospecific metallacyclization [18,19] of terminal alkynes with a low-valent titanium complex, were transformed to π-conjugated polymers containing various building blocks such as 1,3-butadiene, phenylene, 1,4-bismercapto-substituted 1,3-diene, thiophene, selenophene, phosphole, stannole, and tellurophene [20–30]. On the basis of the fact that heteroles of the group 14 and 15 elements such as silole [31,32], stannole [27], phosphole [24,33], and arsole [26] exhibit electron-accepting properties due to the low lowest unoccupied molecular orbital (LUMO) energy levels caused by the σ*-π* orbital interactions between the π* orbitals in the pendant elements-carbon bonds and the π* orbitals of the butadiene units, π-conjugated polymers possessing the corresponding units have been reported to exhibit electron-accepting properties. If the organotitanium polymers possessing electron-donating tethering units could be designed, their transformation reactions would produce π-conjugated polymers containing both electron-donating and electron-accepting alternating units, which serve as ICT π-conjugated polymers. Accordingly, we describe herein the synthesis of π-conjugated polymers containing stannole, phosphole, and arsole units (**5-7**) by the reactions of a titanacyclopentadiene-containing polymer possessing thiophene-2,5-diyl units (**3**). The optoelectronic properties of the obtained π-conjugated polymers are also described.

## 2. Materials and methods

### 2.1. General experimental

^1^H, ^13^C, and ^31^P nuclear magnetic resonance (NMR) spectra were recorded on a JEOL ECP-300 instrument (300 MHz, 75 MHz, and 121 MHz for ^1^H, ^13^C, and ^31^P NMR, respectively). The chemical shift values were expressed relative to tetramethylsilane (TMS) as an internal standard for ^1^H and ^13^C NMR spectra and 85% H_3_PO_4_ as an external standard for ^31^P NMR spectra. Fourier transform infrared (FT-IR) spectra were measured on a Thermo Scientific Nicolet iS10 FT-IR instrument. Size exclusion chromatography (SEC) measurements were performed on a Shimadzu LC-10AS liquid chromatograph equipped with a Shimadzu RID-10A refractive index detector and Tosoh TSK-gel GMHHR-M tandem columns using chloroform (CHCl_3_) as an eluent (1.0 mL/min) at 35°C. UV–vis absorption spectra were recorded in CHCl_3_ on a Shimadzu UV-3100PC spectrometer. Cyclic voltammetric (CV) analyses were carried out on a Versa STAT3 (Princeton Applied Research) potentiostat at a scan rate of 100 mV/s. All the measurements were performed in dry acetonitrile containing 0.10 M tetra-*n*-butylammonium hexafluorophosphate at ambient temperature using a three-electrode system, with each solution being purged with N_2_ prior to measurement. The working electrode was a platinum (Pt) disk (ϕ = 1.6 mm, BAS, Japan), the counter electrode was a Pt wire, and the reference electrode was a silver (Ag) wire.

### 2.2. Materials

2,5-Bis(2-trimethylsilylethynyl)-3-dodecylthiophene [34], and a diethyl ether (Et_2_O) solution of isopropylmagnesium chloride (Pr^*i*^MgCl, 1.0 M) [24] were prepared by previously reported methods. Titanium(IV) isopropoxide [Ti(OPr^*i*^)_4_], dichlorophenylphosphine (PhPCl_2_), and sulfur monochloride (S_2_Cl_2_) were obtained from Sigma-Aldrich, and they were distilled under reduced pressure. Diphenyltin dichloride (Ph_2_SnCl_2_) was obtained from Sigma-Aldrich and recrystallized from hexane. Potassium hydroxide (KOH) was obtained from Sigma-Aldrich. A Et_2_O solution of diiodophenylarsine (PhAsI_2_) was prepared by the reaction of hexaphenylcyclohexaarsine with iodine in Et_2_O [35–37]. Et_2_O was dried over sodium benzophenone ketyl and distilled under nitrogen. The polymerization and the polymer reactions were carried out under argon.

### 2.3 Synthesis of 3-dodecyl-2,5-diethynylthiophene (1)

A mixture of 2,5-bis-trimethylsilylethynyl-3-dodecylthiophene (2.05 g, 4.60 mmol), THF (10 mL), and a methanol (MeOH) solution of KOH (1.0 M, 10 mL) was stirred at ambient temperature for 2 h. Then, the mixture was treated with an aqueous hydrochloric acid solution (1.0 M) and extracted with hexane (50 mL). After drying the combined organics over MgSO_4_, the volatile fractions were evaporated and the residue was purified by silica gel column chromatography (eluent: hexane) to give 2,5-diethynyl-3-dodecylthiophene (**1**) in 88% yield (1.22 g, 4.05 mmol) as a yellow oil.

^1^H NMR (300 MHz, CDCl_3_): 0.85–0.90 (3 H, –C*H*_3_), 1.19–1.38 (18 H, thienyl–(CH_2_)_2_–(C*H*_2_)_9_– CH_3_), 1.51–1.66 (2 H, thienyl – CH_2_–C*H*_2_–), 2.63 (t, *J* = 7.5 Hz, 2 H, thienyl – C*H*_2_–CH_2_–), 3.32 (s, 1 H, –C≡C–*H*), 3.44 (s, 1 H, –C≡C–*H*), 7.01 (s, 1 H); ^13^C NMR (75 MHz, CDCl_3_) 14.1, 22,7, 29.1, 29.3, 29.5, 29.6, 30.0, 31.9, 81.6, 83.9, 118.8, 122.0, 133.7, 148.6; IR (ATR, cm^−1^): 3309, 2923, 2104, 1617, 1528, 1465, 1401, 1377, 1344, 722; HR-MS (FAB): m/z calcd for C_26_H_29_S [M]^+^: 301.1990, found 301.1993.

### 2.4 Synthesis of thiophene-containing polymer (4)

To a dry Et_2_O (20 mL) solution of **1** (0.150 g, 0.500 mmol) and Ti(OPr^*i*^)_4_ (0.199 g, 0.700 mmol) was added a Et_2_O solution of ^*i*^PrMgCl (1.0 M, 1.4 mL, 1.4 mmol) at −78°C under argon, and the mixture was kept stirring at that temperature for 0.5 h and then warmed up to −50°C. After stirring at −50°C for 3 h, S_2_Cl_2_ (0.0810 g, 0.600 mmol) was added at −50°C and the reaction mixture was warmed slowly to ambient temperature. Then, the resulting reaction mixture was poured into an aqueous solution of hydrochloric acid (1.0 M, 50 mL). The organic layer was collected, and the aqueous phase was extracted three times with CH_2_Cl_2_ (total 100 mL). After drying the combined organics over MgSO_4_, the volatile fractions were evaporated and the residue was precipitated into MeOH to give a thiophene-containing polymer (**4**) in 89% yield (0.148 g, 0.445 mmol unit) as a yellow solid.

**4**: ^1^H NMR (300 MHz, CDCl_3_) 0.15–2.96 (25 H, –(C*H*_2_)_11_–C*H*_3_), 6.49–7.81 (aromatic, 3 H); IR (ATR, cm^−1^) 2962, 2926, 2854, 1637, 1560, 1543, 1508, 1458, 1398, 1261, 1095, 1024, 866, 802.

### 2.5 Synthesis of 1,1-diphenylstannole-containing polymer (5)

To a dry Et_2_O (20 mL) solution of **1** (0.150 g, 0.500 mmol) and Ti(OPr^*i*^)_4_ (0.199 g, 0.700 mmol) was added a Et_2_O solution of ^*i*^PrMgCl (1.0 M, 1.4 mL, 1.4 mmol) at −78°C under argon, and the mixture was kept stirring at that temperature for 0.5 h and then warmed up to −50°C. After stirring at −50°C for 3 h, Ph_2_SnCl_2_ (0.206 g, 0.600 mmol) was added at −50°C and the reaction mixture was warmed slowly to ambient temperature. Then, the resulting reaction mixture was precipitated into hexane to give a stannole-containing polymer (**5**) in 77% yield (0.221 g, 0.385 mmol unit) as a red-purple solid.

**5**: ^1^H NMR (300 MHz, CDCl_3_) 0.75–2.68 (25 H, –(C*H*_2_)_11_–C*H*_3_), 7.28–7.88 (aromatic, 13 H); IR (ATR, cm^−1^) 3062, 2946, 2864, 1637, 1660, 1540, 1518, 1458, 1378, 1261, 1195, 1124, 856, 803.

### 2.6 Synthesis of 1-phenylphosphole-containing polymer (6)

To a dry Et_2_O (20 mL) solution of **1** (0.150 g, 0.500 mmol) and Ti(OPr^*i*^)_4_ (0.199 g, 0.700 mmol) was added a Et_2_O solution of ^*i*^PrMgCl (1.0 M, 1.4 mL, 1.4 mmol) at −78°C under argon, and the mixture was kept stirring at that temperature for 0.5 h and then warmed up to −50°C. After stirring at −50°C for 3 h, PhPCl_2_ (0.107 g, 0.600 mmol) was added at −50°C and the reaction mixture was warmed slowly to ambient temperature. Then, the resulting reaction mixture was poured into water (100 mL). The organic layer was collected, and the aqueous phase was extracted three times with dichloromethane (CH_2_Cl_2_, total 100 mL). After drying the combined organics over MgSO_4_, the volatile fractions were evaporated and the residue was precipitated into MeOH to give a phosphole-containing polymer (**6**) in 54% yield (0.111 g, 0.272 mmol unit) as a purple solid.

**6**: ^1^H NMR (300 MHz, CDCl_3_) 0.42–2.91 (25 H, –(C*H*_2_)_11_–C*H*_3_), 6.57–8.03 (aromatic, 8 H); ^13^C NMR (75 MHz, CDCl_3_) 14.1, 22.7, 28.9, 29.4, 29.7, 30.3, 30.6, 31.9, 128.8, 129.3, 131.2, 131.8, 132.2, 134.4, 134.9, 135.0; ^31^P-NMR (121 MHz, CDCl_3_): 6.69; IR (ATR, cm^−1^) 2961, 2921, 2851, 2361, 1456, 1436, 1416, 1259, 1092, 1020, 795, 742.

### 2.7 Synthesis of 1-phenylarsole-containing polymer (7)

To a dry Et_2_O (20 mL) solution of **1** (0.150 g, 0.500 mmol) and Ti(OPr^*i*^)_4_ (0.199 g, 0.700 mmol) was added a Et_2_O solution of ^*i*^PrMgCl (1.0 M, 1.4 mL, 1.4 mmol) at −78°C under argon, and the mixture was kept stirring at that temperature for 0.5 h and then warmed up to −50°C. After stirring at −50°C for 3 h, a Et_2_O solution of PhAsI_2_ (1.0 M, 0.60 mL, 0.60 mmol) was added at −50°C and the reaction mixture was warmed slowly to ambient temperature. Then, the resulting reaction mixture was poured into water (100 mL). The organic layer was collected, and the aqueous phase was extracted three times with CH_2_Cl_2_ (total 100 mL). After drying the combined organics over MgSO_4_, the volatile fractions were evaporated and the residue was precipitated into MeOH to give an arsole-containing polymer (**7**) in 63% yield (0.142 g, 0.314 mmol unit) as a purple solid.

**7**: ^1^H NMR (300 MHz, CDCl_3_) 0.33–2.93 (25 H, –(C*H*_2_)_11_–C*H*_3_), 6.45–8.02 (aromatic, 8 H); ^13^C NMR (75 MHz, CDCl_3_) 14.1, 22.7, 29.4, 29.5, 29.6, 29.7, 30.5, 31.9, 32.1, 129.0, 130.6, 132.7, 133.4, 135.5, 136.7, 140.1, 140.9; IR (ATR, cm^−1^) 2926, 2853, 1579, 1463, 1436, 1095, 825, 735.

## 3. Results and discussion

**Scheme 1.**
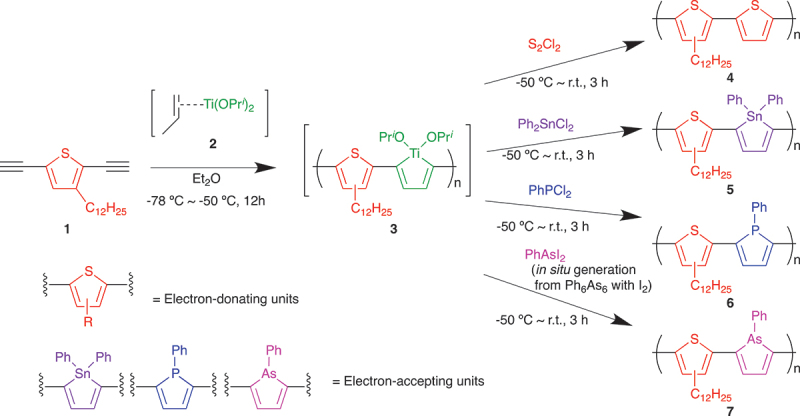
Transformation of **3** to **4**, **5**, **6**, and **7**.

The titanacycropentadiene-containing reactive polymer (**3**) was prepared by the reaction of 2,5-diethynyl-3-dodecylthiophene (**1**) with a titanium(II) complex (**2**) [18,19] generated *in situ* from Ti(OPr^*i*^)_4_ and Pr^*i*^MgCl from −78°C to −50°C in Et_2_O. This polymer (**3**) was subjected to the transformations into polymers containing 1,1-diphenylstannole-2,5-diyl, 1-phenylphosphole-2,5-diyl, and 1-phenylarsole-2,5-diyl units (**5**, **6**, and **7**) by the use of Ph_2_SnCl_2_, PhPCl_2_, and PhAsI_2_, respectively (Scheme 1). For example, the 1,1-diphenylstannole-containing polymer (**5**) was obtained in 77% yield as a red-purple solid by precipitation into MeOH, which is soluble in common organic solvents such as tetrahydrofuran, toluene, CH_2_Cl_2_, and CHCl_3_. The number-average molecular weight (*M*_n_) and the molecular weight distribution (*M*_w_/*M*_n_) of **5** were estimated to be 5,500 and 2.6, respectively, by the SEC. Likewise, the 1-phenylphosphole-containing polymer (**6**: *M*_n_ = 3,000, *M*_w_/*M*_n_ = 1.9) and 1-phenylarsole-containing polymer (**7**: *M*_n_ = 2,500, *M*_w_/*M*_n_ = 2.3) were obtained in 54% and 63% yields, respectively (Table 1). As a reference, a π-conjugated polymer composed solely of the thiophene unit (**4**: *M*_n_ = 7,500, *M*_w_/*M*_n_ = 2.1) was obtained in 89% yield by the reaction of **3** with S_2_Cl_2_. As described in our previous papers, the polymers obtained by these transformations should also have regioregular main chain connections at each heterole unit [23,24,26,27,36]. The structures of **4**, **5**, **6**, and **7** were confirmed by their ^1^H NMR spectra (Figures S3, S4, S5, and S8). In the case of **4**, for example, the relative peak intensities of the aromatic protons at 6.49–7.81 ppm and those of the aliphatic protons at 0.15–2.96 ppm (3.00:28.00) were in accordance with the ratio expected for the proposed structure (3:25). Likewise, the peak intensity ratios of the aromatic and aliphatic protons in **5**, **6**, and **7** agreed well with the ratios expected from the proposed structures. In the ^31^P NMR spectrum of **6**, a broad peak attributable to the phosphole unit was detected at 6.69 ppm (Figure S7). Although the poor S/N ratio of the NMR spectrum caused by the insufficient solubility of **6** makes it difficult to perform an accurate discussion as well as quantitative analysis, a possible minor broad peak at 40 ppm might indicate the presence of the phosphole oxide units [38], which would be produced by the oxidation of the phosphole units during the work-up process.

**Table 1.** Synthesis of **4**, **5**, **6**, and **7** from 3.

Polymers	Yields [%]	*M*_n_ (*M*_w_/*M*_n_)^c)^	Colors
4	89^a)^	7,500 (2.1)	yellow
5	77^b)^	5,500 (2.6)	red-purple
6	54^a)^	3,000 (1.9)	purple
7	63^a)^	2,500 (2.3)	purple

Note: ^a)^ Isolated yield after precipitation into MeOH. ^b)^ Isolated yield after precipitation into hexane. ^c)^ SEC (CHCl_3_, polystyrene Std).

**Figure 1.**
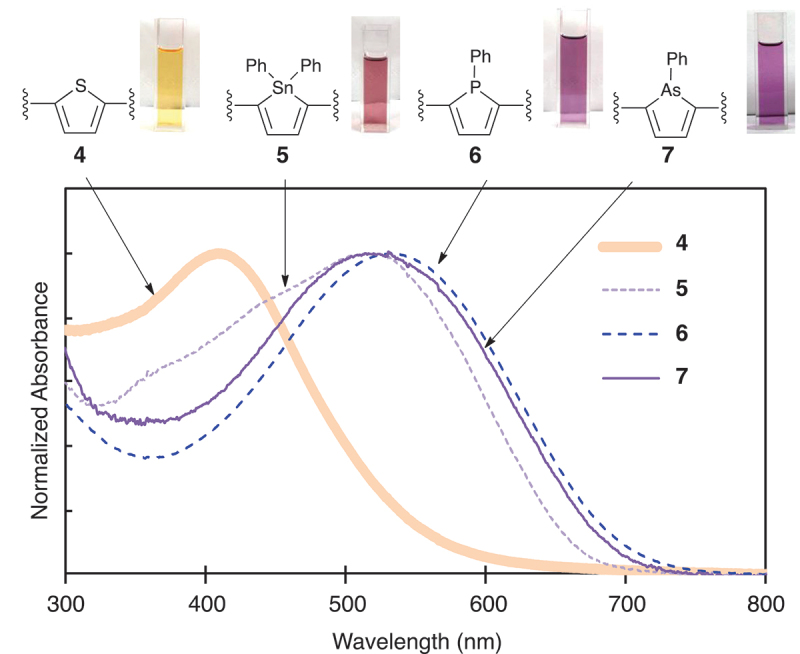
UV-vis absorption spectra of **4**, **5**, **6**, and **7** in CHCl_3_ solutions.

In the UV–vis absorption spectra of **4**, **5**, **6**, and **7** taken in CHCl_3_, the absorption wavelengths were found to be affected by the elements in the heterole units of the polymers (Table 2 and Figure 1). That is, the absorption maxima (λ_max_) of the thiophene-, stannole-, phosphole-, and arsole-containing polymers (**4**, **5**, **6**, and **7**) were observed at 410 nm, 514 nm, 536 nm, and 530 nm, respectively. The optical band gaps (*E*_g(opt)_) estimated from the absorption onsets (λ_onset_) of the polymers (**4**, **5**, **6**, and **7**) were 2.25 eV, 1.86 eV, 1.78 eV, and 1.79 eV, respectively. It was clearly demonstrated that the polymers (**5**, **6**, and **7**) possessing both the electron-donating and electron-accepting building blocks exhibit narrower band gap compared to the case of the polymer (**4**). The optical band gap of the polymers was found to decrease in the order of **4** > **5** > **7** > **6**. These differences are due to the different electronic properties of the heteroles incorporated in the polymers, which could be supported by their CV analyses.

**Table 2.** Optical properties of **4**, **5**, **6**, and **7**.

Polymers	_λmax_^a)^ [nm]	_λonset_^a)^ [nm]	*E*_g(opt)_^b)^ [eV]
4	410	550	2.25
5	514	668	1.86
6	536	697	1.78
7	530	692	1.79

Note: ^a)^ Measured in CHCl_3_; ^b)^ Estimated from λ_onset_.

**Figure 2.**
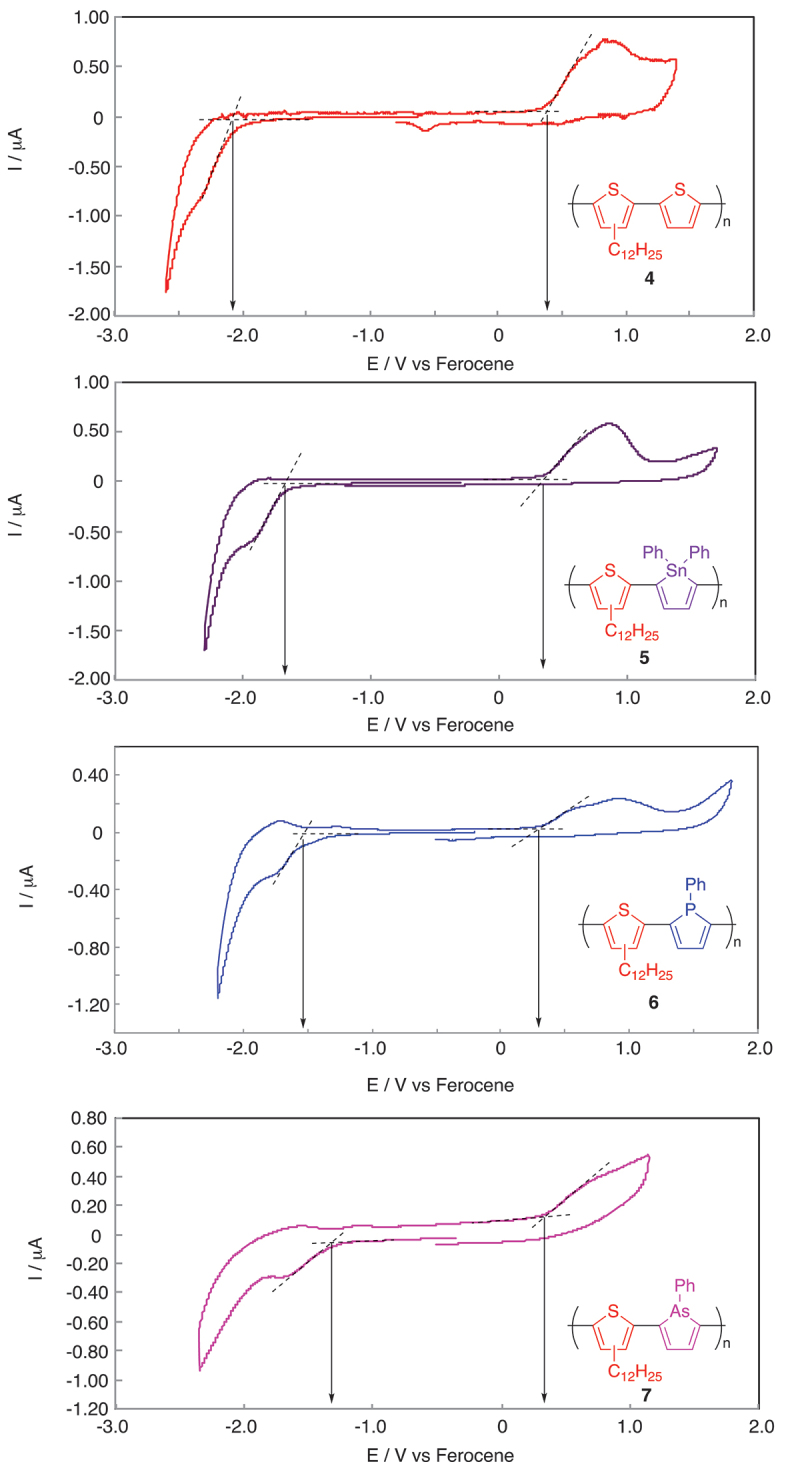
Cyclic voltammograms of **4**, **5**, **6**, and **7** in film on Pt disks immersed in acetonitrile solutions containing tetra-*n*-butylammonium hexafluorophosphate (0.10 M), at a sweep rate of 100 mV s^−1^.

The oxidation and reduction potentials of the polymers (**4**, **5**, **6**, and **7**) were estimated from their CV analyses (Figure 2). The LUMO energy levels were estimated from the onsets of oxidation and reduction peaks, where *E*_ox_ and *E*_red_ are the onset potentials of oxidation and reduction, respectively, observed in the CV analyses (Table 3) [39]. All the polymers (**4**, **5**, **6**, and **7**) exhibit high highest occupied molecular orbital (HOMO) energy levels originated from the electron-rich thiophene unit. These values are higher than those of the corresponding phenylene-containing polymers by approximately 0.3 eV, as reported in our previous papers [24,26,27]. The LUMO energy levels of the polymers (**5**, **6**, and **7**) are lower than that of **4** due to the low-lying LUMO energy levels of stannole, phosphole, and arsole rings caused by the σ*-π* orbital interactions between the heteroatoms (Sn, P, and As) and the diene unit as supported by the DFT calculations [24,26,27]. Accordingly, the polymers (**5**, **6**, and **7**) exhibit the narrower band gap, originated from the high HOMO and low LUMO energy levels of the alternating heterocyclic structures.

**Table 3.** Electrochemical properties of **4**, **5**, **6**, and **7**.

Polymers	*E*_ox_ (V)^a)^	*E*_red_ (V)^a)^	HOMO (eV)^b)^	LUMO (eV)^c)^
4	0.34	−2.06	−5.14	−2.74
5	0.34	−1.66	−5.14	−3.14
6	0.33	−1.55	−5.13	−3.25
7	0.33	−1.50	−5.13	−3.30

Note: ^a)^ Estimated from cyclic voltammetric analyses (film, immersed in acetonitrile). ^b)^ *E*(HOMO) = -(*E*_ox_ +4.80) (eV), where *E*_ox_ is the onset potential of oxidation, observed in the cyclic voltammetric analyses. ^c)^ *E*(LUMO) = -(*E*_red_ +4.80) (eV), where *E*_red_ is the onset potential of reduction.

## 4. Conclusions

A regioregular reactive organometallic polymer having titanacyclopentadiene-2,5-diyl and tethering electron-rich thiophene units in the main chain (**3**) was prepared by the reaction of 2,5-diethynyl-3-dodecylthiophene (**1**) and a titanium(II) complex (**2**) generated *in situ* from Ti(OPr^*i*^)_4_ and Pr^*i*^MgCl from −78°C to −50°C in diethyl ether. The organotitanium polymer (**3**) was subjected to the transformation reactions without isolation to produce 1,1-diphenylstannole-containing, 1-phenylphosphole-containing, and 1-phenylarsole-containing polymers (**5**, **6**, and **7**) by reactions with diphenyltin dichloride, dichlorophenylphosphine, and diiodophenylarsine, respectively. The resulting polymers (**5**, **6**, and **7**) exhibit ICT properties because they are composed of the alternating electron-rich thiophene and electron-deficient heteroles generated by the polymer reactions. Applications of the polymers to organic devices such as OSCs are now in progress.

## Supplementary Material

Supplemental Material
