# Supplementary material for: Parallel synthesis of donor-acceptor π-conjugated polymers by post-element transformation of organotitanium polymer
Source: Des Monomers Polym. 2023 Jul 6;26(1):190–7. doi: 10.1080/15685551.2023.2233228 (PMC10327520; doi:10.1080/15685551.2023.2233228)
Supplement: Supplemental Material [file TDMP_A_2233228_SM5798.pdf]

Supplementary information for

Parallel synthesis of donor-acceptor π-conjugated polymers by post-element-transformation of organotitanium polymer

Yoshimasa Matsumura,[a,c] Alvin Tanudjaja,[a] Mizuki Fukushima,[a] Makoto Higuchi,[a] Shin Ogino,[a] Makoto Ishidoshiro,[b] Yasuyuki Irie,[b] Hiroaki Imoto,[b] Kensuke Naka,[b] Ryoyu Hifumi,[a] Shinsuke Inagi,[a] and Ikuyoshi Tomita[a,]*

[a] Department of Chemical Science and Engineering, Graduate School of Materials and Chemical Technology, Tokyo Institute of Technology, Nagatsuta-cho 4259-G1-9, Midori-ku, Yokohama 226-8502, JAPAN

[b] Graduate School of Science and Technology, Kyoto Institute of Technology, Goshokaido-cho, Matsugasaki, Sakyo-ku, Kyoto 606-8585, JAPAN

[c] Present Address: Department of Applied Chemistry, Faculty of Engineering, Osaka Institute of Technology, 5-16-1 Omiya, Asahi-ku, Osaka 535-8585, JAPAN

* E-mail: tomita@cap.mac.titech.ac.jp

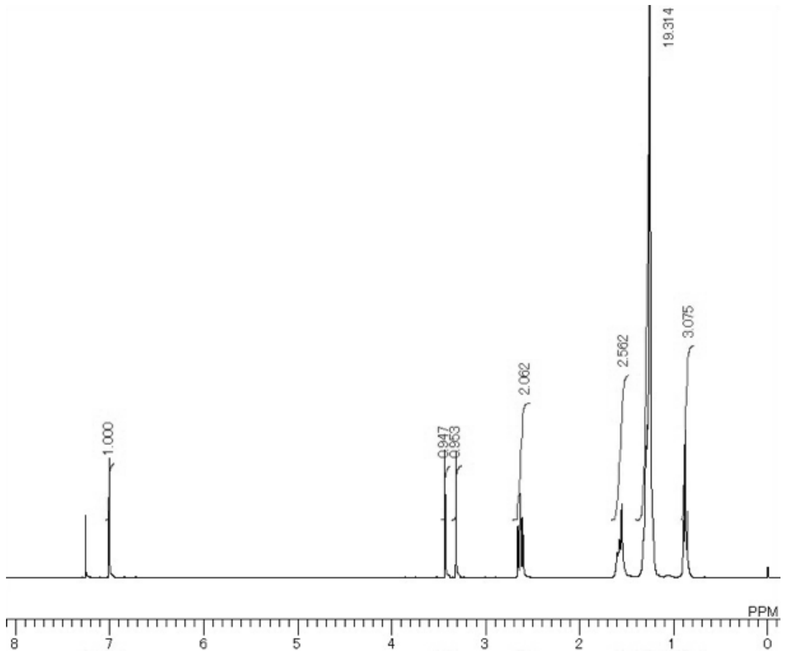

Figure S1. ^{1}H NMR spectrum (300 MHz) of 2,5-diethynyl-3-dodecylthiophene (**1**) in $CDCl_3$.

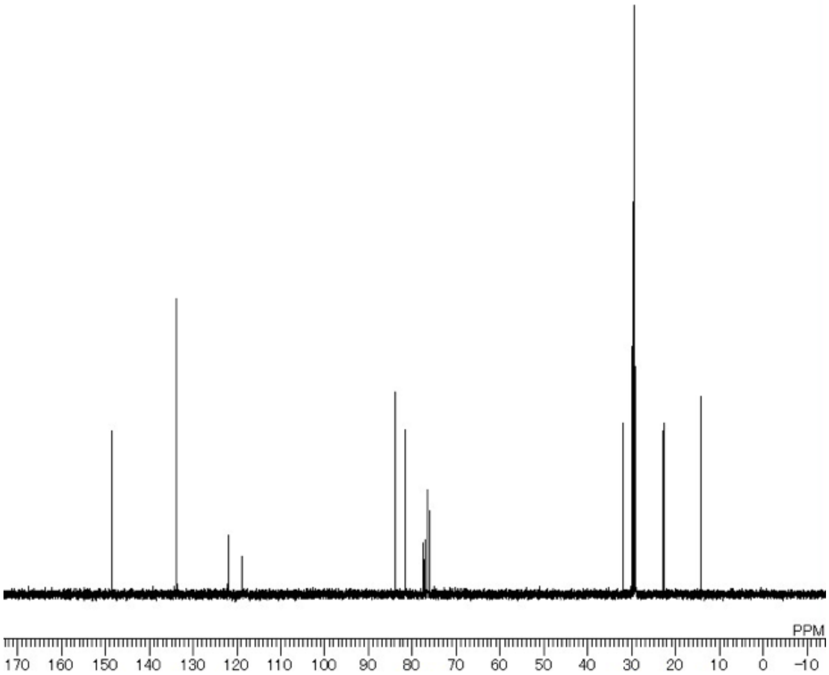

Figure S2. ^{13}C NMR spectrum (75 MHz) of 2,5-diethynyl-3-dodecylthiophene (**1**) in $CDCl_3$.

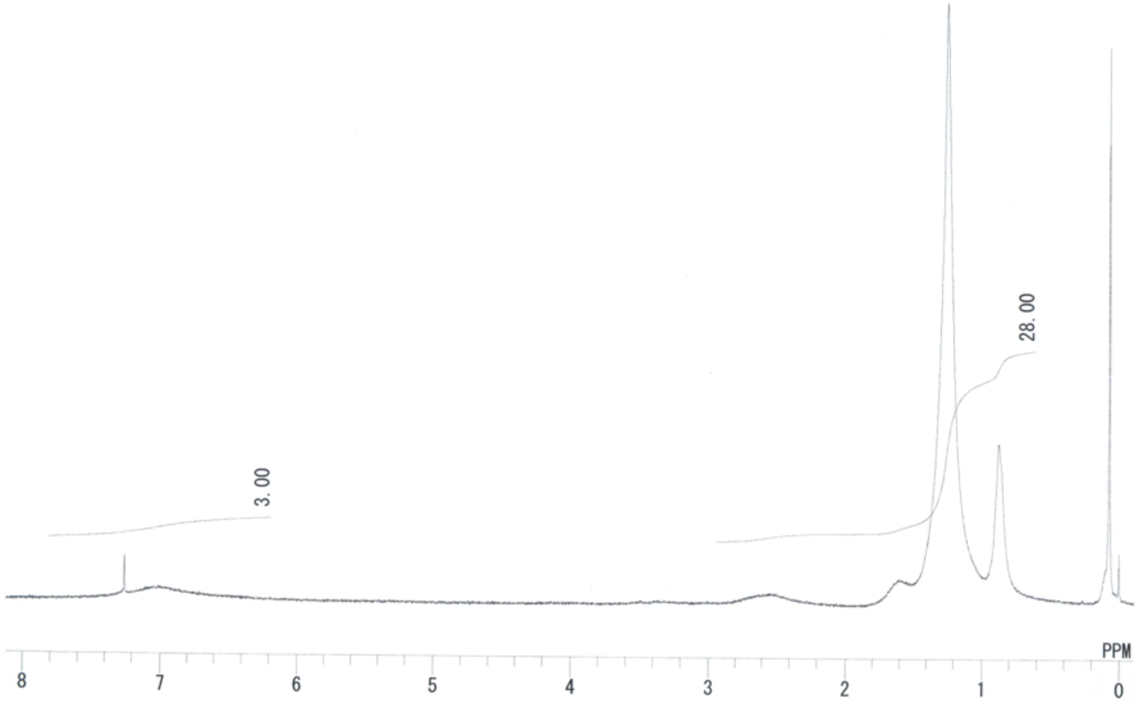

Figure S3. ^{1}H NMR spectrum (300 MHz) of a thiophene-containing polymer (**4**) in $CDCl_3$.

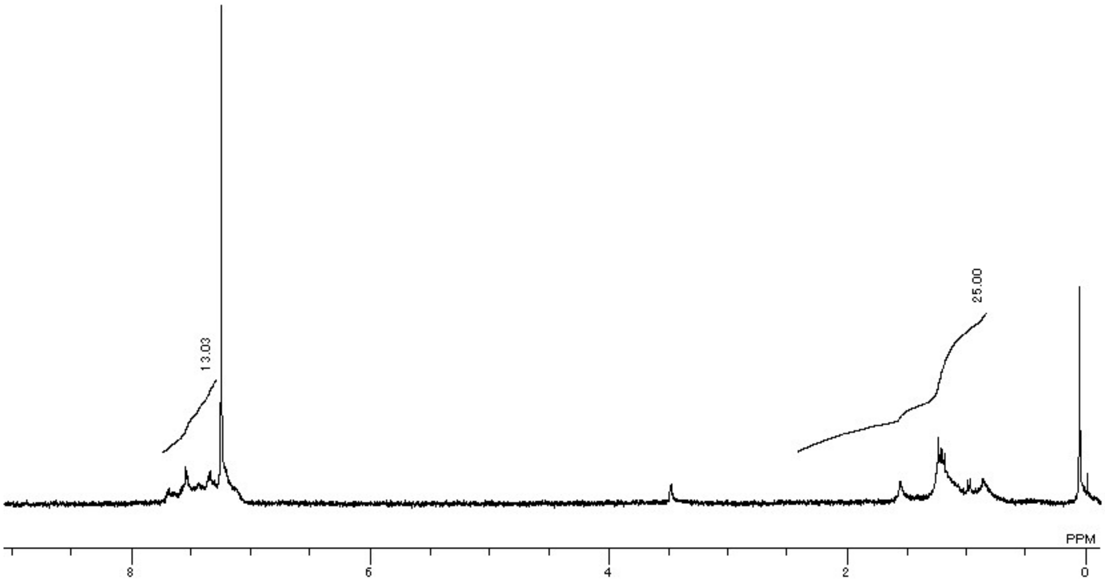

Figure S4. ^{1}H NMR spectrum (300 MHz) of a 1,1-diphenylstannole-containing polymer (**5**) in $CDCl_3$.

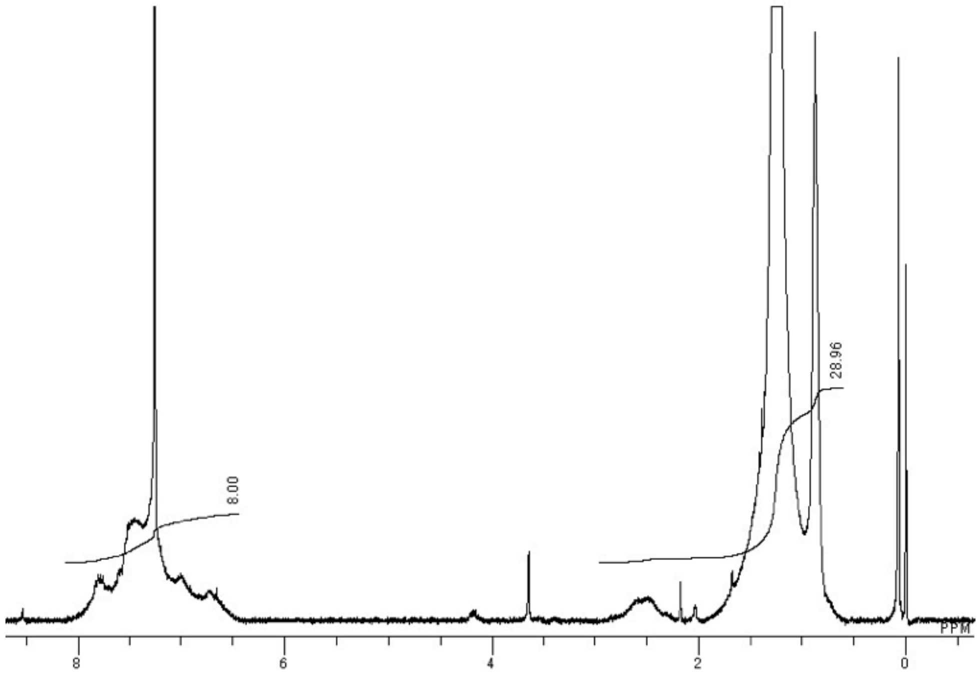

Figure S5. ^{1}H NMR spectrum (300 MHz) of a 1-phenylphosphole-containing polymer (**6**) in $CDCl_3$.

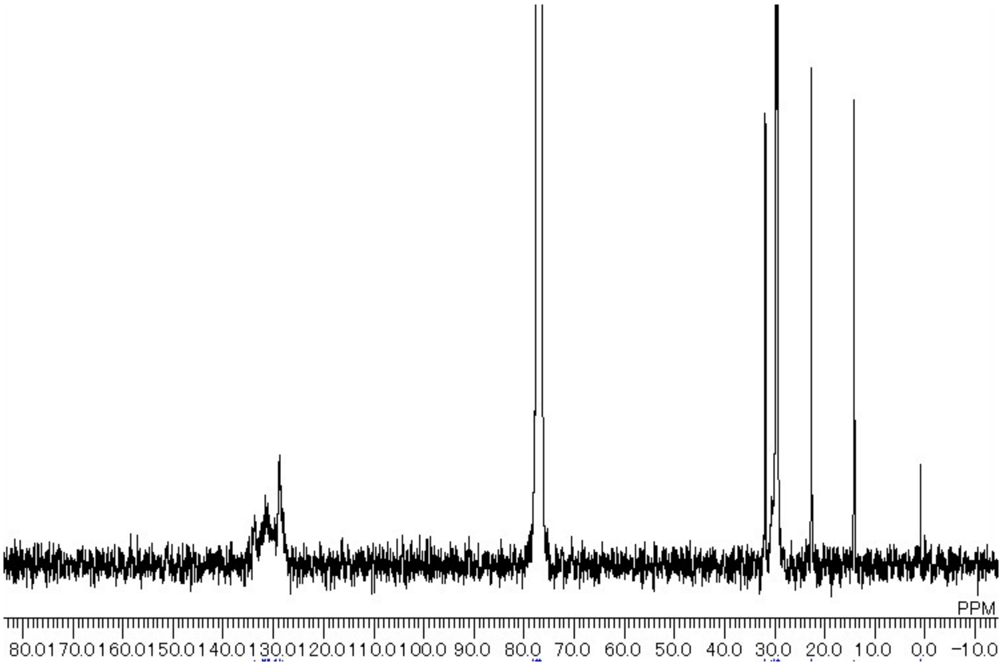

Figure S6. ^{13}C NMR spectrum (75 MHz) of a 1-phenylphosphole-containing polymer (**6**) in $CDCl_3$.

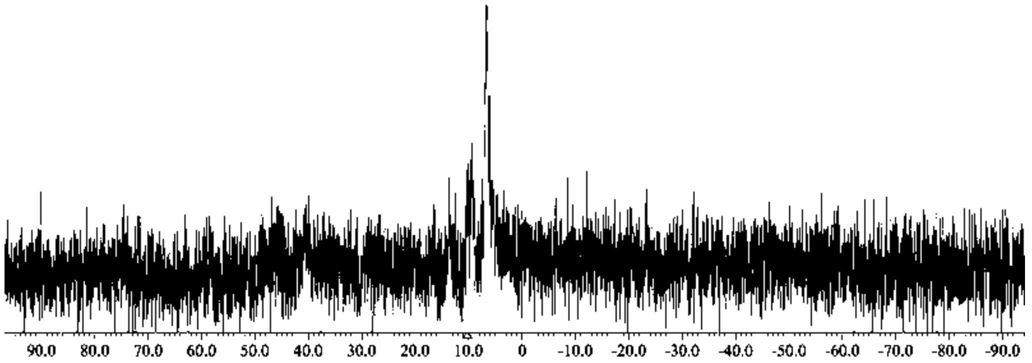

Figure S7. ^{31}P NMR spectrum (121 MHz) of a 1-phenylphosphole-containing polymer (**6**) in $CDCl_3$.

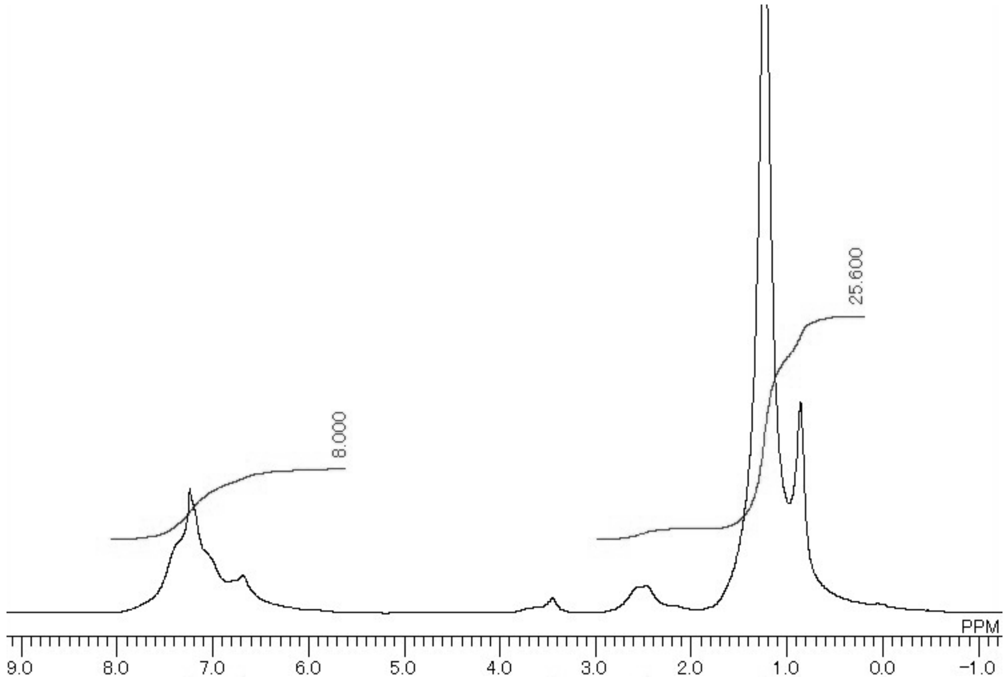

Figure S8. ^{1}H NMR spectrum (300 MHz) of a 1-phenylarsole-containing polymer (**7**) in $CDCl_3$.

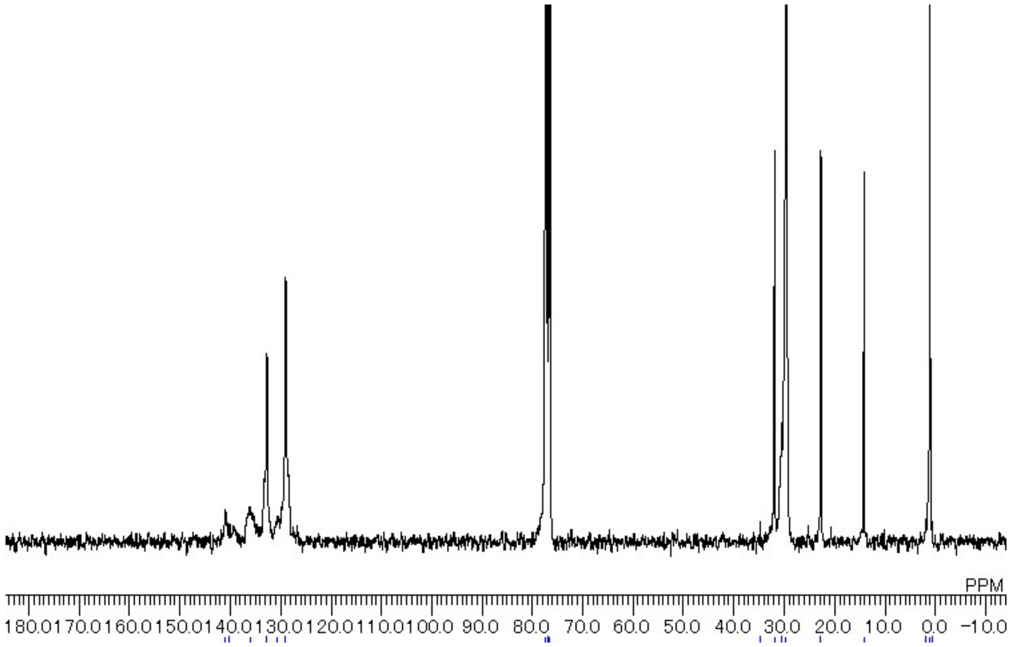

Figure S9. ^{13}C NMR spectrum (75 MHz) of a 1-phenylarsole-containing polymer (**7**) in $CDCl_3$.
